# Global serum proteomic changes in water buffaloes infected with *Fasciola gigantica*

**DOI:** 10.1186/s13071-019-3533-5

**Published:** 2019-06-03

**Authors:** Fu-Kai Zhang, Rui-Si Hu, Hany M. Elsheikha, Zhao-An Sheng, Wei-Yu Zhang, Wen-Bin Zheng, Xing-Quan Zhu, Jun-Jun He

**Affiliations:** 1 State Key Laboratory of Veterinary Etiological Biology, Key Laboratory of Veterinary Parasitology of Gansu Province, Lanzhou Veterinary Research Institute, Chinese Academy of Agricultural Sciences, Lanzhou, 730046 Gansu People’s Republic of China; 2 Faculty of Medicine and Health Sciences, School of Veterinary Medicine and Science, University of Nottingham, Sutton Bonington Campus, Loughborough, LE12 5RD UK; 3 College of Animal Science and Technology, Guangxi University, Nanning, 530005 Guangxi Zhuang Autonomous Region People’s Republic of China; 4 Jiangsu Co-innovation Center for Prevention and Control of Important Animal Infectious Diseases and Zoonoses, Yangzhou, 225009 Jiangsu People’s Republic of China

**Keywords:** *Fasciola gigantica*, iTRAQ, PRM, Coagulation, Complement, Lysozyme, Buffalo

## Abstract

**Background:**

The liver fluke *Fasciola gigantica* modulates several signaling pathways in infected buffaloes to facilitate its survival and establishment of persistent infection. In response to the parasite invasion, buffaloes activate innate and adaptive immune responses to counter the parasite infection. To detect new proteins that might be involved in the interaction between *F. gigantica* and the buffaloes, and that also might serve as biomarkers for fasciolosis, we used proteomic techniques to study the serum proteome of buffaloes during *F. gigantica* infection. Here, we used an isobaric tags for relative and absolute quantitation (iTRAQ)-based quantitative proteomic approach to identify serum proteins that are differentially expressed in infected buffaloes compared to uninfected control buffaloes. Additionally, we applied a parallel reaction monitoring (PRM) assay to validate specific proteins identified by the iTRAQ method.

**Results:**

A total of 313, 459 and 399 proteins were identified at 3, 42 and 70 days post-infection, respectively; of these 92, 93 and 138 were differentially abundant proteins. Some of the identified differentially abundant proteins, including complement factor H related 5, complement component C6, complement component C7, amine oxidase, plasma serine protease inhibitor and lysozyme, are known to be involved in complement system activation, blood coagulation, platelet activation, lymphocyte’s adhesion and lysozyme hydrolysis. Analysis of data for all three time points after infection identified six significantly upregulated proteins in infected serum that separated infected and uninfected buffaloes into distinct clusters. Further PRM analysis confirmed the expression of five proteins, namely MHC class I antigen, Beta-2-microglobulin, NID2 protein, Fetuin-B and Fibrinogen gamma-B chain.

**Conclusions:**

These findings provide novel insights into the serum proteomics signature of buffaloes during *F. gigantica* infection.

**Electronic supplementary material:**

The online version of this article (10.1186/s13071-019-3533-5) contains supplementary material, which is available to authorized users.

## Background

Fasciolosis, caused by infection with *Fasciola gigantica* or *F. hepatica*, is an important parasitic disease in tropical and temperate regions, respectively [1, 2]. Ungulates and humans become infected *via* ingestion of contaminated water or plants containing metacercariae, leading to adverse health consequences [3]. Fasciolosis can cause significant economic losses in the agricultural industry [4]. The impact on human health is enormous, with ~180 million people at risk of infection and 17 million people infected [5]. *Fasciola* infection has been associated with liver fibrosis, cirrhosis and cancer in affected individuals [6] and liver flukes have been reported in the bile duct of humans [7–9]. Current methods to control liver fluke infection rely on the use of fasciocidal drugs such as triclabendazole. However, increasing anthelmintic resistance has become a major concern [10] and together with the lack of a commercial vaccine, makes control of liver fluke infection challenging [11]. In order to develop better therapeutic strategies that can successfully control fasciolosis, it is important to have a full understanding of the molecular mechanisms involved in *Fasciola* interactions with host effector systems.

Other key aspects for efficient liver fluke control includes offering veterinarians access to better diagnostic tools which allow a prompt and accurate detection of infection. In this respect, having novel biomarkers may add value to current diagnostic tests and improve clinical decision making by enabling earlier detection of *Fasciola* infection. Therefore, identification of infection-specific molecular changes in buffaloes can reveal new biomarkers and advance the understanding of the pathogenesis of *F. gigantica* infection. Transcriptomic data have been obtained from buffalo livers infected with *F. gigantica* [12]; however, mRNA abundance does not necessarily correlate with protein abundance [13]. The cytokine dynamics of the buffalo’s serum during *F. gigantica* infection has been investigated [14]. However, cytokines appeared to have a limited diagnostic value on their own because they are general products of the host systemic inflammatory response to cell injury or infection.

In recent years, proteomic technology has developed rapidly and has been widely applied to identify biomarkers in studies, for example, on myocardial infarction [15], tuberculosis [16] and *F. hepatica* infection [17]. Global proteomic studies are, therefore, of great importance for understanding the pathophysiology that underpins infection states, including infection with *F. gigantica*. The isobaric tags for relative and absolute quantification (iTRAQ) is one of the most sensitive proteomics techniques currently used to compare protein expression across different biological conditions [18–20] and infection status [21]. A traditional limitation of proteomics techniques is the inability to confirm the identity of specific proteins. Interestingly, parallel reaction monitoring (PRM) is a new mass spectrometry method that is more specific and sensitive than traditional mass spectrometry methods and can detect target proteins [22].

In this study, an iTRAQ-based quantitative proteomic analysis of serum of *F. gigantica*-infected buffaloes was performed to explore dynamic serum protein responses of buffaloes towards *F. gigantica* infection. A set of five differentially abundant proteins were further validated by PRM analysis. By performing this combined quantitative proteomic analysis of serum from *F. gigantica*-infected *versus* uninfected buffaloes (control), we found that *F. gigantica* infection alters several key biological processes and specific proteins. Our findings provide a global overview for the buffalo serum responses to *F. gigantica* infection and highlight new targets for further investigation.

## Methods

### Preparation of encysted metacercariae

Eggs of *F. gigantica* were collected from the gall bladder of naturally infected buffaloes slaughtered at local abattoirs in Guangxi Zhuang Autonomous Region, PR China. The collected eggs were incubated at 29 °C for 11 days. The newly hatched miracidia were used to infect *Galba pervia* snails (3–5 miracidia per snail) maintained in plastic trays for 2 h. The infected snails were incubated in order to allow the miracidia to develop to sporocysts, rediae and cercariae. After ~6 weeks, cercariae were shed from infected snails and harvested on 5 × 5 cm cellophane sheets in order to form metacercariae. Encysted metacercariae harvested on cellophane sheets were washed several times with phosphate buffered saline (PBS) and used to infect buffaloes as described previously [23].

### Animals and experimental infection

Twenty-four (8–10-month-old) buffaloes were purchased from a water buffalo farm in Guangxi Zhuang Autonomous Region, PR China. Animals were randomly divided into two groups: (i) uninfected, control group (12 buffaloes); and (ii) infected group (12 buffaloes). Animals within each group were assigned to 4 subgroups (3 buffaloes per group). To ensure that all buffaloes used in the study were free of any liver fluke infection, fecal samples were examined by sedimentation technique for detection of fluke eggs and sera were tested for anti-*F. gigantica* IgG and IgM antibodies using ELISA, as described previously [24]. Additionally, buffaloes were treated with triclabendazole (1 ml, 5% per kilogram BW). After four weeks withdrawal time, 12 buffaloes in the infected group were infected orally with 500 viable metacercariae per animal, whereas control animals were mock-inoculated with 0.85% NaCl solution without metacercariae [12].

### Serum preparation and depletion of high abundance proteins

At 3, 42 and 70 days post-infection (dpi), whole blood samples of all animals in each group were collected aseptically into tubes without anticoagulant. Samples were allowed to clot and stored in a cooler on ice for up to 4 h post-collection before being centrifuged at 2500×*g* for 10 min. Serum was collected and stored at −80 °C until use. Livers were collected after slaughter of animals and examined visually for gross pathological lesions. Highly-abundant proteins were depleted using the ProteoMiner™ protein enrichment kit (Bio-Rad, Hercules, CA, USA) following the manufacturer’s instructions and protein concentration was determined by using a Bradford assay. Approximately 100 μg of protein of each sample was digested with Trypsin Gold (Promega, Madison, WI, USA) at a ratio of 1:50 (trypsin to protein, wt/wt) at 37 °C overnight (~16 h). Digested peptides were desalted with a C18 cartridge to remove the high urea, and the desalted peptides were dried by vacuum centrifugation.

### iTRAQ labeling of peptides

Desalted peptides were labeled with iTRAQ reagents (iTRAQ^®^ Reagent-8PLEX Multiplex Kit; Sigma, St. Louis, MO, USA) according to the manufacturer’s instructions. For 100 μg of peptides, 1 unit of labeling reagent was used. Peptides were dissolved in 20 μl of 0.5 M triethylammonium bicarbonate (TEAB) pH 8.5 solution and labeling reagent was added in 70 μl of isopropanol. After 1 h of incubation, the reaction was stopped with 100 μl of 50 mM Tris-HCl pH 8. Differentially labeled peptides were mixed equally and then desalted in peptide desalting spin columns (Thermo Fisher Scientific, Waltham, MA, USA).

### High performance liquid chromatography (HPLC) fractionation

The iTRAQ-labeled peptide mix was fractionated using a Durashell RP column (5 μm, 100 Å, 250 × 4.6 mm; Agela, Tianjin, China) on a Rigol L3000 HPLC (RIGOL SCIENTIFIC, Allerød, Denmark) operating at 1 ml/min. Mobile phases A (2% acetonitrile, 20 mM NH4FA, adjusted pH to 10 using NH_3_·H_2_O) and B (80% acetonitrile, 20 mM NH4FA, adjusted pH to 10 using NH_3_·H_2_O) were used to develop a gradient elution. The tryptic peptides were separated at an eluent flow rate of 1 ml/min and monitored at 214 nm (UV). The column oven was set as 37 °C. Eluent was collected every minute. The samples were dried under vacuum and reconstituted in 15 μl of 0.1% (v/v) formic acid (FA) in water for subsequent analysis.

### LC-MS/MS analysis

Shotgun proteomics analyses were performed using an EASY-nLC^TM^ 1200 UHPLC system (Thermo Fisher Scientific) coupled to an Orbitrap Fusion Lumos mass spectrometer (Thermo Fisher Scientific) operating in the data-dependent acquisition (DDA) mode. A sample volume corresponding to 2 μg of total peptides reconstituted in 0.1% FA was injected onto an Acclaim PepMap100 C18 Nano-Trap column (2 cm × 100 μm, 5 μm; Dionex, Sunnyvale, USA). Peptides were separated on a Reprosil-Pur 120 C18-AQ analytical column (15 cm × 150 μm, 1.9 μm; Dr. Maisch HPLC GmbH, Ammerbuch-Entringen, Germany) using a 75 min linear gradient from 5 to 100% eluent B (0.1% FA in 80% acetonitrile) in eluent A (0.1% FA in H_2_O) at a flow rate of 600 nl/min. For DDA, the Orbitrap Fusion Lumos mass spectrometer was operated in positive polarity mode with spray voltage of 2.3 kV and capillary temperature of 320 °C. Full mass spectrometry (MS) scans from 300 to 1500 m/z were acquired at a resolution of 60,000 resolving power (at 200 m/z) with an AGC target value of 4 × 105 and a maximum ion injection time of 50 ms. The MS2 scans were acquired at a resolution of 15,000 resolving power (at 200 m/z) with an automatic gain control (AGC) target value of 5 × 104, a maximum ion injection time of 35 ms, and a normalized collision energy of 36%.

### Protein identification

The MS raw data files (.wiff files) obtained from Triple-TOF 5600 were submitted to ProteinPilot v.4.2 using the Paragon search engine against the *Bos taurus* protein database. To reduce the probability of false peptide identification, we counted only peptides at a cutoff value of 95% confidence interval by a ProteinPilot probability analysis greater than “identity” as identified, and each confident protein identification was supported by at least one unique peptide. Proteome Discoverer v.2.1 software was used to compare the quantitative data. We used ratios with *P*-values ≤ 0.05, and fold changes of ≥1.2 or ≤0.83 were considered as significant.

### Functional prediction

Three databases were used to predict gene and protein functions. These were GO (Gene Ontology, http://www.geneontology.org) [25], KEGG (Kyoto Encyclopedia of Genes and Genomes, http://www.genome.jp/kegg/) [26, 27] and COG (Clusters of Orthologous Groups, http://www.ncbi.nlm.nih.gov/COG/) [28]. Fisher’s exact test followed by FDR correction [29] was performed to identify significantly enriched GO terms or pathways. The FDR corrected *P*-value < 0.05 was used as the threshold of significantly enriched.

### Verification of proteomic results using parallel reaction monitoring (PRM)

A PRM assay allows sensitive and rapid analysis of preselected proteins [30]. Therefore, PRM was applied to additional samples in order to verify protein expression patterns obtained by iTRAQ-based proteomic analysis. Briefly, protein preparation was performed as described above. Targeted MS analysis using PRM was performed on a TripleTOF 5600+ LC-MS/MS system (AB SCIEX, Concord, ON). ProteinPilot software was used to identify proteins and peptide precursor ions for the DDA mode acquired raw data, and the database search results were analyzed using Skyline software v.4.2 to obtain a rough spectra library. Target proteins for PRM validation were imported into Skyline, and the peptides for protein quantification were selected according to the ion signals. The PRM method was run against the biological samples of interest, evaluated and refined to develop the highest quality assay. Data processing was performed in Skyline, and the results of the quantification were manually inspected for each peptide of the targeted proteins.

## Results

### Confirmation of *F. gigantica* infection

At 70 dpi, livers collected from infected animals showed clear gross pathological lesions together with the presence of adult *F. gigantica* flukes, confirming the establishment of infection in all experimentally infected buffaloes. By contrast, livers of uninfected control buffaloes appeared normal, without any pathological changes and free of *F. gigantica* flukes.

### Proteomics profiles of different groups

**Fig. 1:**
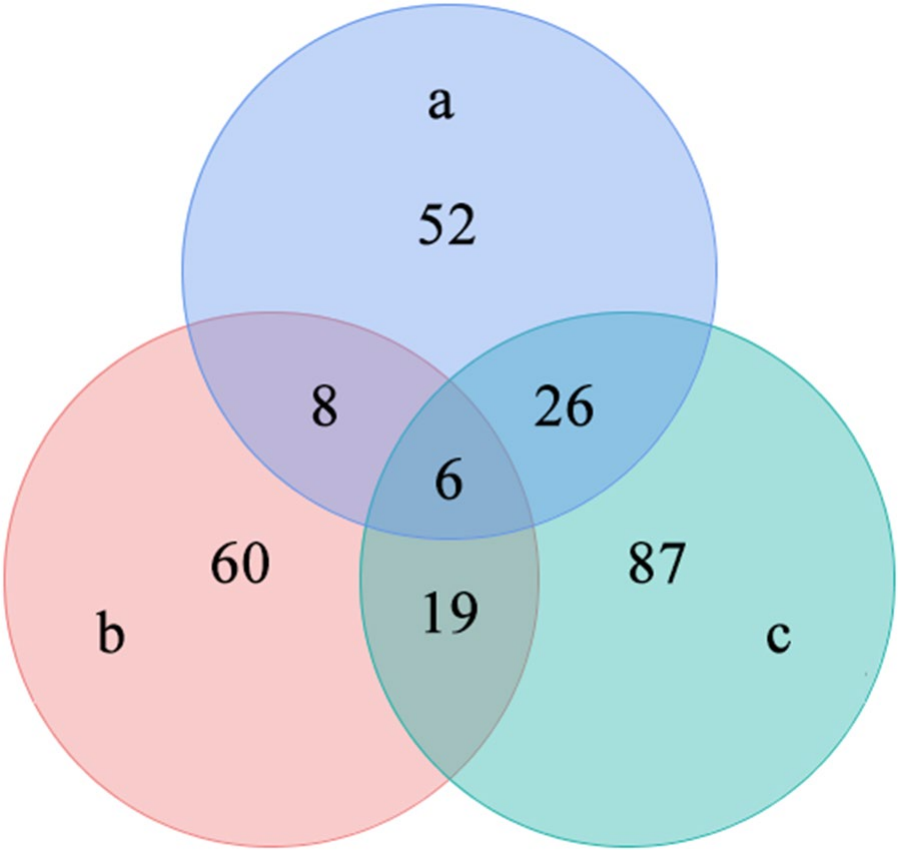
Venn diagram showing the unique and shared differentially expressed serum proteins between *Fasciola gigantica*-infected animal groups at 3 (**a**), 42 (**b**) and 70 (**c**) days post-infection

**Fig. 2:**
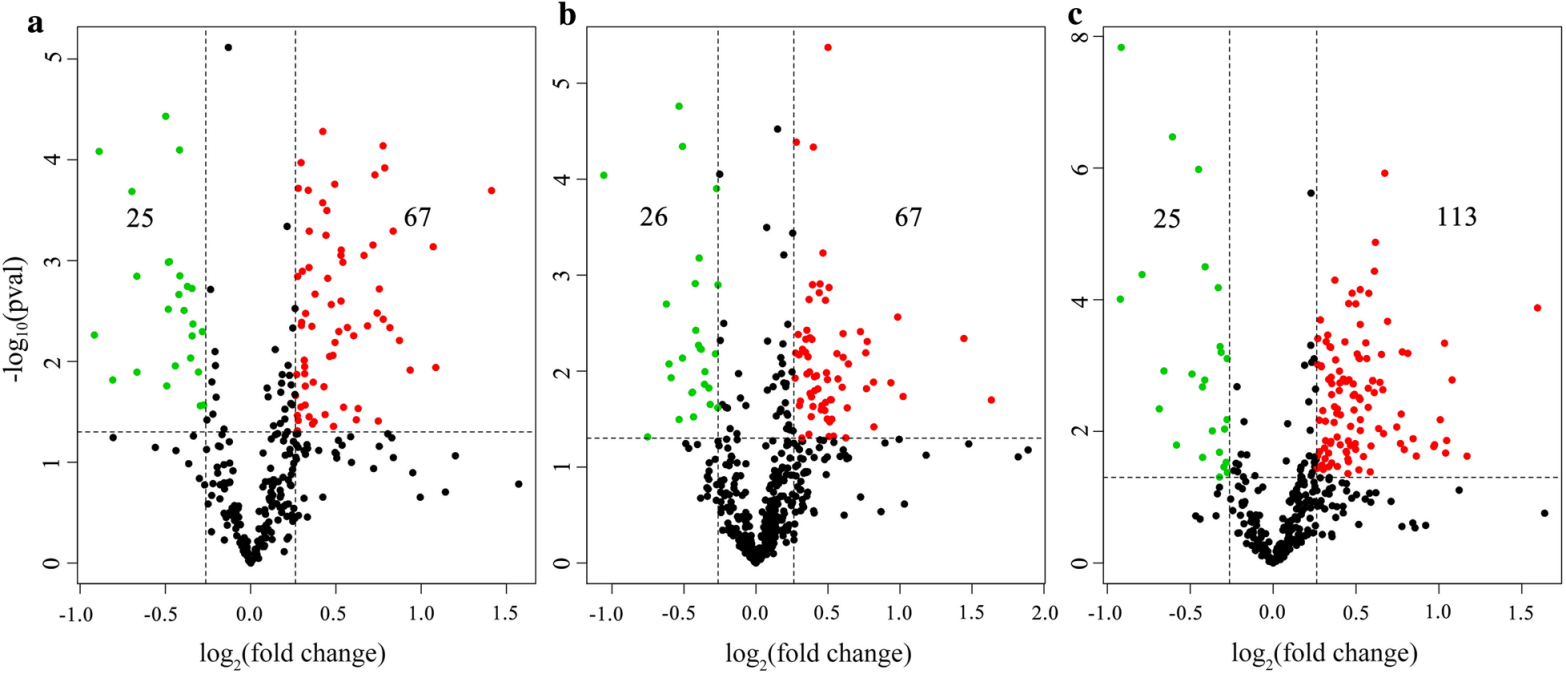
Volcano plots showing the differentially expressed proteins detected in the serum of *F. gigantica*-infected compared to uninfected control buffaloes. The most statistically significant proteins are shown toward the top, with upregulated proteins in red dots and downregulated proteins in green dots. Black dots represent no significant differences. The x-axis represents log_2_(fold change) values and y-axis represents −log_10_(pval) values. **a**, **b** and **c** represent differentially expressed proteins at 3, 42 and 70 days post-infection, respectively

Proteomics analysis revealed 313, 459 and 399 serum proteins at 3, 42 and 70 dpi, respectively. Compared to control, 67, 67 and 113 proteins were significantly upregulated at 3, 42 and 72 dpi, respectively. However, a total of 25, 26 and 25 proteins showed significantly low expression levels in infected samples that were collected at 3, 42 and 72 dpi, respectively (Figs. 1, 2 and Additional file 1: Table S1). E1BCJ2, E1BD43, F1MM86, F1N045, F2X047 and Q9N2I2 were the most commonly differentially expressed protein at all time points after infection (3, 42 and 72 dpi).

### PRM validation of the protein expression in buffalo serum

**Fig. 3:**
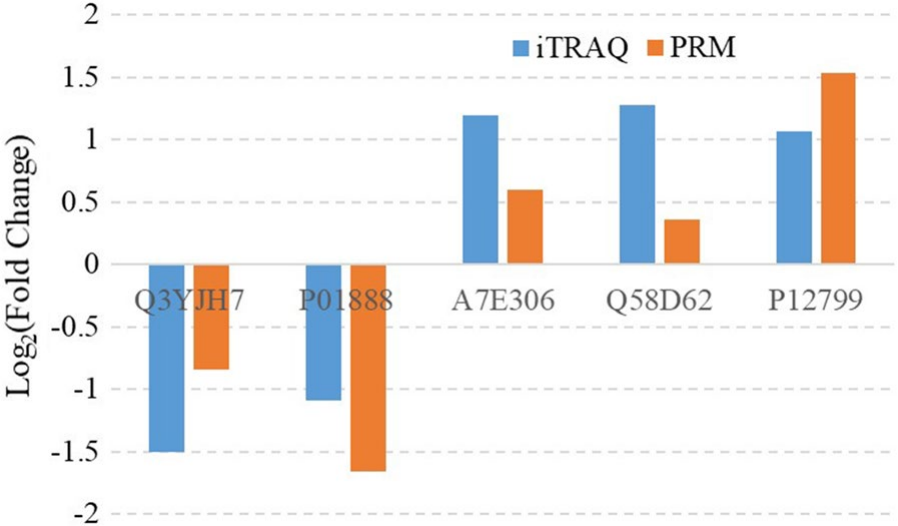
PRM verification of proteins identified by iTRAQ analysis. Five proteins were selected for validation of the iTRAQ data. Data of iTRAQ were verified by PRM at 3 (**a**), 42 (**b**) and 70 (**c**) days post-infection, respectively

Five significantly expressed proteins, including MHC class I antigen (UniProt identifier Q3YJH7), Beta-2-microglobulin (UniProt identifier P01888), NID2 protein (UniProt identifier A7E306), Fetuin-B (UniProt identifier Q58D62) and Fibrinogen gamma-B chain (UniProt identifier P12799), were selected randomly and their expression was examined by PRM. The trends of the level of expression of these five proteins obtained by iTRAQ and PRM were similar (Fig. 3).

### Cluster of Orthologous Groups of proteins (COG) analysis

**Fig. 4:**
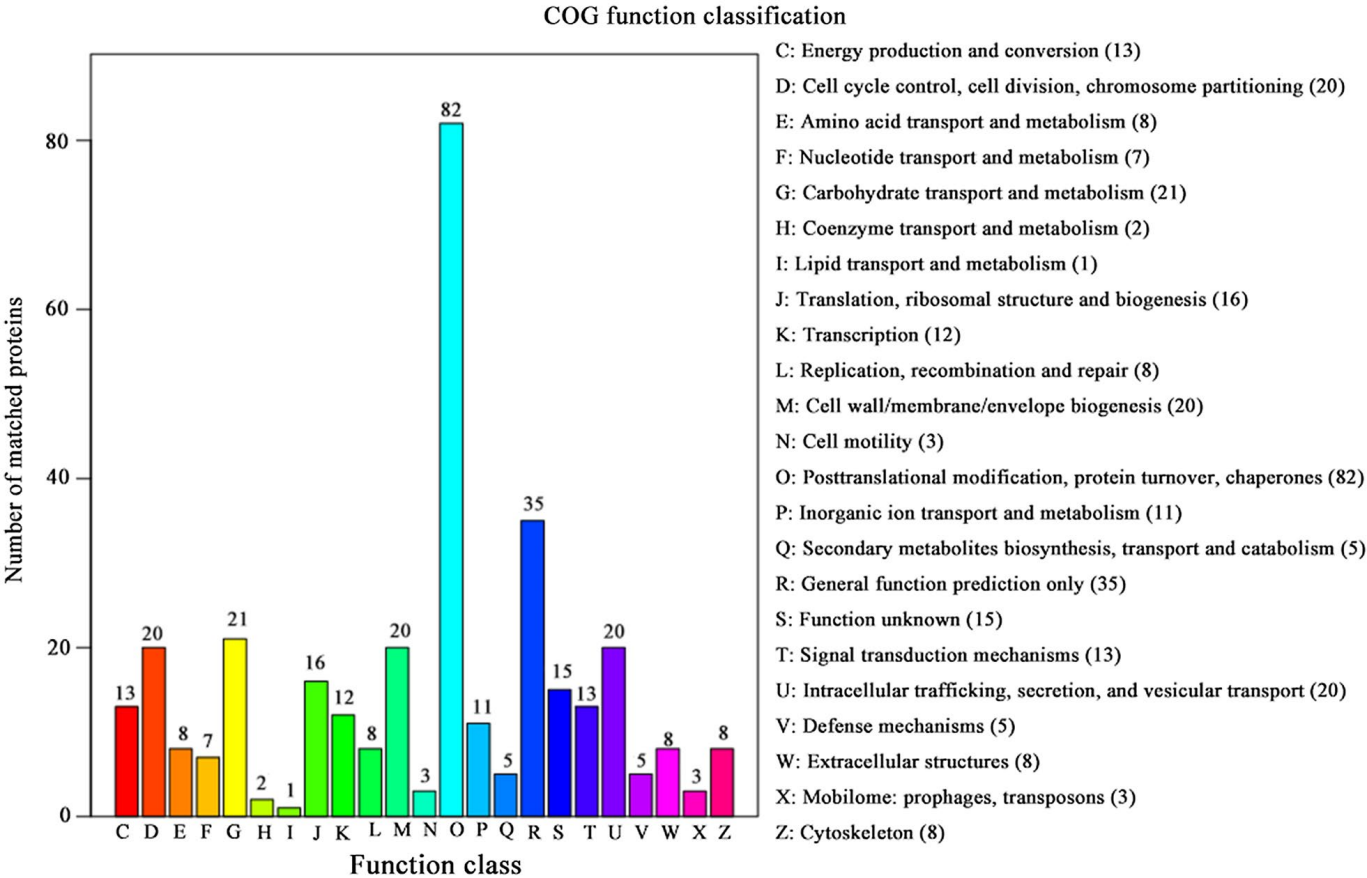
COG function classification. The x-axis represents the function’s class and the y-axis shows the number of matched proteins

All identified proteins were classified into 23 COG clusters including energy production and conversion, cell cycle control, cell division, chromosome partitioning, and amino acid transport and metabolism. Among these, matched proteins in “posttranslational modification, protein turnover, chaperones” accounted for the largest proportion in all COG. Matched proteins in COG function classification are shown in Fig. 4.

### GO classification and KEGG analysis

**Fig. 5:**
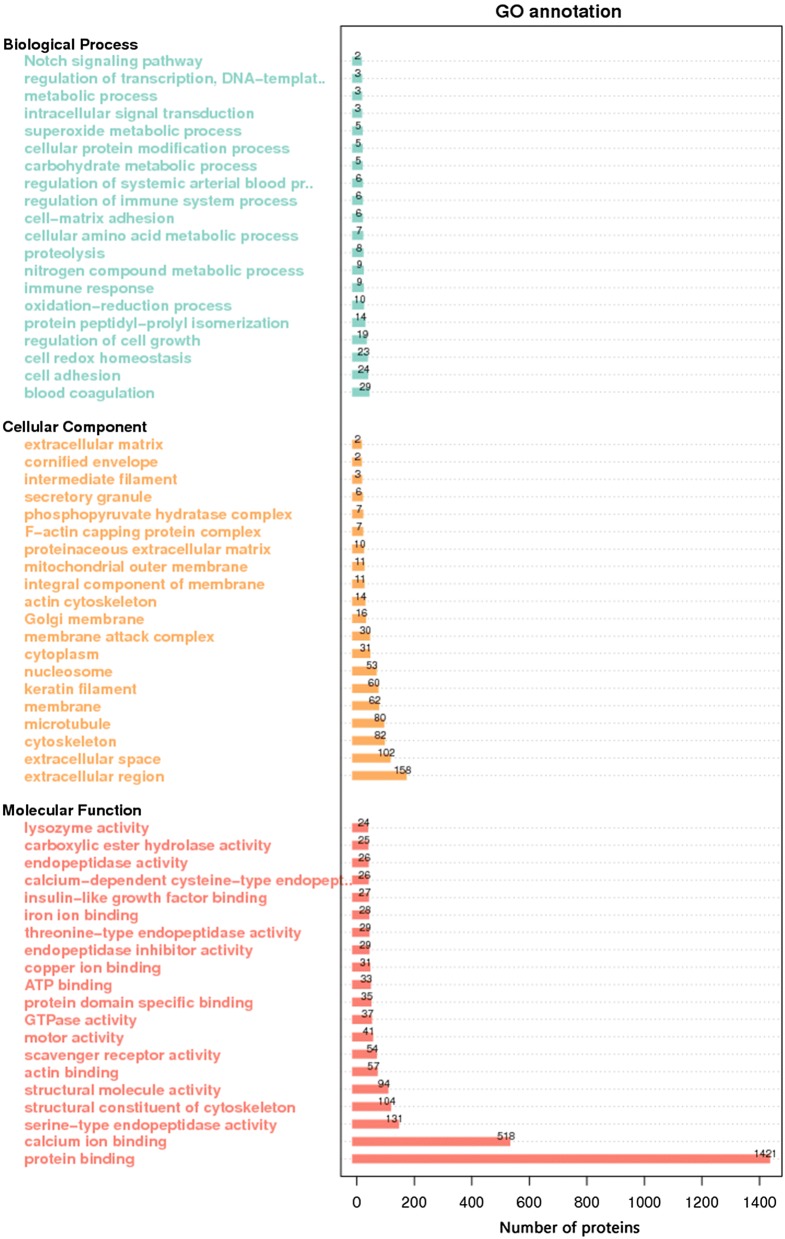
GO annotation of all identified proteins. Identified proteins in all three groups were stratified into three categories: molecular function, cellular component and biological process

**Fig. 6:**
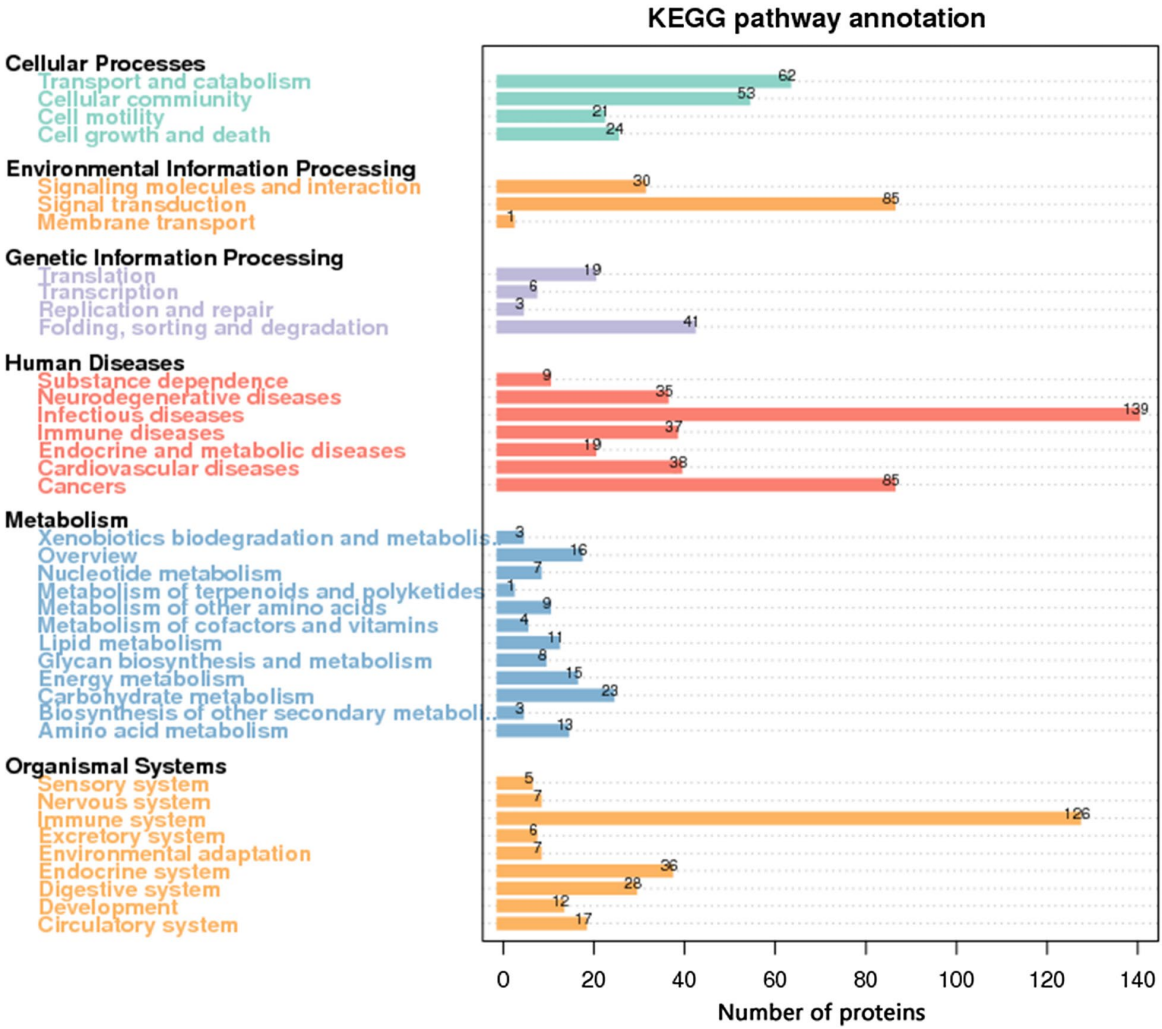
Statistics of KEGG pathway annotation. The x-axis shows the number of proteins; the y-axis corresponds to KEGG pathway annotation

GO classification was performed to study the biological function of the identified proteins in the buffalo sera. The identified proteins were classified into “molecular function”, “biological process” and “cellular component” as shown in Fig. 5. Most proteins were involved in molecular functions that are essential for biological activities. According to KEGG analysis, 6 sections containing 39 pathways were identified in the buffalo sera as shown in Fig. 6. “signal transduction” in “environmental information processing”, “infectious diseases” and “cardiovascular diseases” in “human diseases”, and “immune system” in “organismal systems” were significantly highly enriched compared to other maps. Most of the differentially expressed proteins were enriched in immune system and infection diseases.

### Heatmap clustering of identified proteins in buffalo serum

**Fig. 7:**
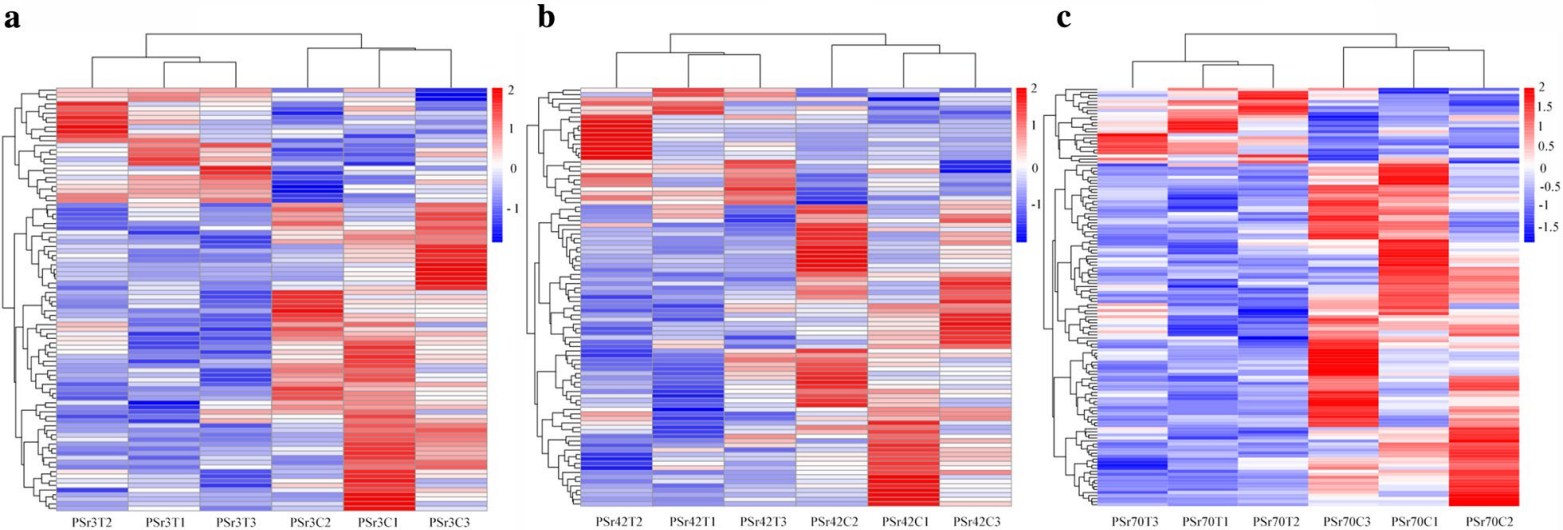
Heatmaps of the differentially expressed proteins. **a**, **b** and **c** show the differentially expressed proteins at 3, 42 and 70 days post-infection, respectively. The red (upregulated) and blue (downregulated) colors represent the differentially expressed proteins; the white color represents the proteins whose expression levels were not different from control, uninfected samples

All identified proteins were clustered according to the expression data. As shown in Fig. 7, at each time point the infected samples and control samples were clustered into different branches. The identified proteins at each time point had significantly different expression pattern between control and infected group.

## Discussion

The mechanisms whereby *F. gigantica* influences the serum proteome of buffaloes have not yet been fully elucidated. In this study, we employed the iTRAQ technique to uncover the global serum proteomic changes in buffaloes infected with *F. gigantica*. Our results showed that the serum protein profiles of infected buffaloes and non-infected buffaloes were clearly separated (Fig. 7). In the present study, *F. gigantica* influenced the expression of serum proteins associated with protein binding, immune systems and signal transduction. Our iTRAQ-based proteomics analysis revealed 92, 93 and 138 differentially expressed proteins at 3, 42 and 70 dpi, respectively (Additional file 1: Table S1 and Fig. 2). iTRAQ results were validated by re-testing the expression of five proteins using PRM. Results obtained by iTRAQ had a similar expression trend with PRM (Fig. 3), suggesting the reliability of our iTRAQ analysis.

After newly excysted juveniles emerge in the duodenum of the definitive host, they migrate across the peritoneum to the liver and eventually to the bile duct. During this journey juvenile flukes feed on the host blood and undergo maturation to egg-laying adult flukes [31]. Additionally, they must adapt to different host microenvironments and engage in various biological processes, such as blood coagulation and complement activation. In the following sections, we discuss the influence of *F. gigantica* on blood coagulation, complement activation, and other immune response mechanisms in buffalo serum.

### *Fasciola gigantica* influences coagulation and platelet activation

Protein C inhibitor (PCI or SERPINA5) participates in many biological progresses including its diverse roles in blood coagulation as a pro- and anti-coagulant factor [32]. At 3 dpi, PCI was significantly upregulated compared to control buffaloes. Due to its dual roles in coagulation, we can hypothesize that *F. gigantica* utilizes PCI’s anti-coagulant property instead of pro-coagulant ability in order to ensure enough blood for the feeding of juvenile flukes. The downregulation of C5 and upregulation of PCI at 42 dpi suggest that *F. gigantica* reduces coagulation of buffalo blood to maintain enough food for the development of the growing flukes. At 70 dpi, PCI expression was reduced, suggesting that chronic *F. gigantica* infection may enhance the host coagulation process. These findings indicate that there are two distinct processes involved in host coagulative responses to fluke infection and that those mechanisms may be differentially affected by stage of infection.

Platelets play an essential role in the hemostatic process, and not only contribute to the formation of the primary plug, but also accelerate coagulation [33]. Prior research has shown that several classes of surface glycoproteins are essential for primary platelet responses [33] and different proteins and glycoproteins are expressed at the surface of *F. gigantica*. Thus, there is a reason to believe that *F. gigantica* can manipulate the platelet activation pathway in buffaloes during infection. P12799 (fibrinogen gamma chain, gene name: FGG) was downregulated at 70 dpi. Together with fibrinogen alpha (FGA) and fibrinogen beta (FGB), FGG polymerizes to form an insoluble fibrin matrix [34], which is one of the primary components of blood clots [35]. Furthermore, FGG encoding protein functions during early stages of wound repair to stabilize the lesion and guide cell migration during re-epithelialization [36]. The downregulation of FGG can reduce the formation of blood clots and platelet aggregation in buffaloes infected with *F. gigantica*, which may make blood more accessible to the flukes. On the other hand, upregulation of F1MAV0, F1MDH3, P60712, P61223 and Q32LP0 can lead to platelet activation. Therefore, excess platelets and less clotting may be one of the mechanism that underpin hemorrhagic anaemia in hosts infected with liver flukes

### The complement system and *F. gigantica* infection

E1BCJ2 (gene name: CFHR5, complement factor H related 5), E1BD43 (gene name: AOC3, protein name: amine oxidase involved in lymphocyte binding), F1MM86 (gene name: C6; protein name: complement component C6 involved in the regulation of complement cascade), F1N045 (gene name: C7; protein name: complement component C7), F2X047 (gene name: LYZ; protein name: lysozyme involved in hydrolysis signaling pathway), and Q9N2I2 (gene name: SERPINA5; protein name: plasma serine protease inhibitor) were differentially expressed in sera of infected buffaloes at all three time points after infection.

Previous studies have investigated the role of Th2 [12], Th1/Th17 [23], cytokines [14] and antibody [37] responses in the immunopathogenesis of *F. gigantica*. However, reports about complement component changes during *F. gigantica* infection are limited. This is the first report, to our knowledge, of the involvement of buffalo complement components at different phases of *F. gigantica* infection. F1MM86 (C6) and F1N045 (C7) with C5b, C8 (F1MX87) and multiple copies of C9 (Q3MHN2) are complement components of the membrane attack complex (MAC), which is known to compromise osmotic integrity and induce cell death in pathogens [38]. F1MM86 and F1N045 showed similar trends during infection with significantly high expression at early (3 dpi) and late (70 dpi) infection. A previous study showed that a *F. gigantica-*specific antibody that promotes the activation of complement cascade pathway was increased from 3 weeks post-infection (wpi) and peaked at 13 wpi [37]. This result suggests that *F. gigantica* can modulate the buffalo’s immune responses, especially the complement system to promote its survival and persistence in the liver of buffaloes. The influence of *F. hepatica* on the complement activation pathways has been also reported [39].

The classical pathway of complement activation is part of the adaptive immune response and is initiated with antibody binding [40]. The lectin pathway and the alternative pathway are two complement pathways, which are independent of antibody-antigen interaction [41]. The lection pathway is initiated by carbohydrates on microbial surfaces [42, 43] and the alternative pathway can be initiated by blood-coagulation [44]. Given the upregulation of F1MM86 and F1N045 at 3 and 70 dpi, we infer that lectin and carbohydrate on the surface of *F. gigantica*, and specific host antibody can promote the complement responses at 70 dpi. This is the first report of the influence of *F. gigantica* on the complement in buffaloes.

### *Fasciola gigantica* affects recruitment of lymphocytes

Vascular adhesion protein-1 (VAP-1, also called amine oxidase copper containing-3 [AOC3]), is an endothelial cell adhesion molecule that promotes recruitment of lymphocytes, such as CD8^+^ T cells, CD4^+^ T cells and NK cells to the liver [45]. The granulocyte adhesion to VAP-1 is mediated by Siglec-9 [46, 47]. VAP-1 is constitutively expressed in the sinusoids of human liver and is activated during an immune response [48], especially in inflammatory liver disease [49]. The expression of VAP-1 has been also associated with various tumors, such as astrocytoma [50], colorectal cancer [51], gastric cancer [52] and breast cancer [53].

At 3 and 42 dpi, *F. gigantica* induces an inflammatory response and enhances VAP-1 homing of lymphocytes to liver, but in the meantime the fluke attenuates inflammatory responses by downregulating lipopolysaccharide binding protein (LBP) to promote its own survival [12]. At 70 dpi, there was a significant downregulation of VAP-1 in the buffalo sera. It is possible that VAP-1 plays roles that can be compensated for by other molecules. For example, activated immune responses mediated by TLRs and NLRs were previously reported in buffalo livers at 70 dpi with *F. gigantica* [23]. These findings indicate that in the course of *F. gigantica* infection, coordinated pro- and anti-inflammatory signals allow the flukes to evade the host immune control and prevent excessive inflammatory response from inducing extensive tissue damage in order to favor parasite persistence.

### Lysozyme hydrolyzes glycosphingolipids in *F. gigantica*

The upregulation of lysozyme at 3, 42 and 70 dpi indicates that buffaloes may activate lysosome in response to *F. gigantica* as part of the antiparasitic defense strategies to damage the fluke’s structural integrity *via* targeting glycosphingolipids. Lysozymes are key players of the innate immune system and possess antimicrobial activity [54]. The lysozyme catalyzes the hydrolysis of 1,4-beta-linkage between N-acetylmuramic acid and N-acetyl-D-glucosamine in peptidoglycans and between the N-acetyl-D-glucosamine residues in chitodextrins [55]. A previous study found no link between lysozyme and *F. hepatica* [56]. However, *F. hepatica* expresses the globo-series of glycosphingolipids [Gal(α1-4)Gal(β1-4)Glc(β1-1)Cer] [57] and glycosphingolipids isolated from *F. hepatica* and *F. gigantica* [58] are a subtype of glycolipids, containing the amino alcohol sphingosine, and there is a 1,4-beta linkage between galactose and glucose. The presence of a 1,4-beta linkage between these two molecules indicate that lysozyme may hydrolyze glycosphingolipids in *F. gigantica* and *F. hepatica*.

## Conclusions

In the present study, the iTRAQ-based proteomics technique coupled with a parallel reaction monitoring approach was used to identify the differentially abundant proteins in the serum of *F. gigantica*-infected *versus* control buffaloes. A total of 313, 459 and 399 proteins were identified at 3, 42 and 70 days post-infection, respectively; of these 92, 93 and 138 were differentially abundant proteins. The most significant differentially abundant protein markers that distinguished sera of infected from uninfected buffaloes included complement factor H related 5, complement component C6, complement component C7, amine oxidase, plasma serine protease inhibitor and lysozyme, which are known to be involved in complement system activation, blood coagulation, platelet activation, lymphocyte adhesion and lysozyme hydrolysis. Further characterization of buffalo proteins involved in the pathogenesis of *F. gigantica* may lead to the discovery of novel therapeutic or diagnostic targets.

## Additional file

**Additional file 1: Table S1.** Differentially expressed proteins identified in the present study.

## Data Availability

The data supporting the findings of this article are included within the article and its Additional files. The mass spectrometry proteomics data have been deposited at the ProteomeXchange Consortium *via* the PRIDE partner repository with the dataset identifier PXD011576.

## Abbreviations

iTRAQ: isobaric tags for relative and absolute quantitation
PRM: parallel reaction monitoring
dpi: days post-infection
MAC: membrane attack complex
VAP-1: vascular adhesion protein-1
LBP: lipopolysaccharide binding protein
